# Adrenal suppression in children treated with swallowed fluticasone and oral viscous budesonide for eosinophilic esophagitis

**DOI:** 10.1186/s13223-016-0154-9

**Published:** 2016-10-10

**Authors:** Alexandra Ahmet, Eric I. Benchimol, Ellen B. Goldbloom, Janice L. Barkey

**Affiliations:** 1 Division of Endocrinology, Children’s Hospital of Eastern Ontario, 401 Smyth Rd, Ottawa, ON K1H 8L1 Canada; 2 Division of Gastroenterology, Hepatology and Nutrition, Children’s Hospital of Eastern Ontario, Ottawa, ON Canada; 3 Children’s Hospital of Eastern Ontario Research Institute, Ottawa, ON Canada; 4 Department of Pediatrics, University of Ottawa, Ottawa, ON Canada; 5 School of Epidemiology, Public Health and Preventive Medicine, University of Ottawa, Ottawa, ON Canada

**Keywords:** Adrenal suppression, Children, Cohort studies, Cortisol, Diagnostic tests, Eosinophilic esophagitis, Glucocorticoid, Treatment

## Abstract

**Background:**

Adrenal suppression (AS), a glucocorticoid (GC) side effect associated with significant morbidity, is well described related to inhaled corticosteroid therapy for asthma. Swallowed topical glucocorticoid therapy is the main pharmacotherapy treatment for eosinophilic esophagitis (EoE) and therefore children with EoE are potentially at increased risk of AS.

**Methods:**

In this prospective cohort study, we included children and youth <18 years diagnosed with EoE and treated with swallowed fluticasone or oral viscous budesonide for more than 1 month. First morning cortisol and low dose adrenocorticotropic hormone stimulation test (LDST) were performed 2 weeks following GC discontinuation. AS was defined as an abnormal LDST result (cortisol peak <500 nmol/L). We determined the prevalence and duration of AS related to swallowed topical GC therapy in EoE by LDST, as well as the diagnostic accuracy of first morning cortisol compared to LDST.

**Results:**

Of 29 participants enrolled, 26 (89.7 %) received oral viscous budesonide and 3 (10.3 %) received swallowed fluticasone. Nineteen (65.5 %) participants had AS. Median duration of AS was 43 weeks. Five (17.2 %) participants had persistent AS at 12 months. There were no identifiable risk factors for the development of AS. First morning cortisol was highly specific but had poor sensitivity for detection of AS.

**Conclusions:**

The majority of children with EoE had AS after discontinuation of swallowed topical GC therapy. Stress steroids should be considered in children treated with swallowed topical GC therapy for EoE, even after GC discontinuation, to prevent possible adrenal crisis.

**Electronic supplementary material:**

The online version of this article (doi:10.1186/s13223-016-0154-9) contains supplementary material, which is available to authorized users.

## Background

Adrenal suppression (AS), a potential side effect of glucocorticoid (GC) therapy, is the most common form of secondary adrenal insufficiency [1]. Significant morbidity and mortality related to AS in patients treated with inhaled corticosteroids (ICS) has been well described in the asthma population [2, 3].

The swallowed portion of ICS that escapes first pass inactivation by the liver may lead to systemic side effects [4]. Swallowed fluticasone or viscous budesonide are the mainstays of pharmacotherapy in eosinophilic esophagitis (EoE) [5]. Children treated for EoE are therefore potentially at increased risk of AS and its associated morbidity. Guidelines exist for screening and management of potential AS in children treated with ICS for asthma [6]. However there are currently no guidelines addressing the risk in children receiving swallowed ICS for EoE.

AS occurs due to negative feedback inhibition when the hypothalamic-pituitary-adrenal axis is exposed to exogenous GCs, resulting in decreased cortisol production [7]. Symptoms of AS are often non-specific and may go unrecognized until an adrenal crisis is precipitated by physiologic stress such as illness, surgery, injury, or anesthesia [1]. Adrenal crisis presents as hypotension, hypoglycemia, shock, decreased consciousness, seizure or even death [7] and has been well described related to GC therapy for asthma and malignancy in children [2, 8]. AS can persist for months to years following discontinuation of GC therapy [9, 10]. Prevention of adrenal crisis requires recognition of AS and administration of GCs during times of physiological stress [1].

We conducted a prospective cohort study to determine the prevalence and duration of AS in children with EoE. In addition, we determined whether first morning cortisol was a sensitive and specific screening test for AS in the population, or whether a low dose ACTH stimulation test (LDST) was necessary to make the diagnosis. We also sought to determine risk factors for the development of AS, and the diagnostic accuracy of first morning cortisol as a screening tool for the diagnosis of AS in children with EoE.

## Methods

### Study design

We conducted a prospective observational cohort study in the EoE clinic at the Children’s Hospital of Eastern Ontario (CHEO), Ottawa, Canada.

The study was approved by the Research Ethics Board of the Children’s Hospital of Eastern Ontario (CHEO REB Protocol No: 11/181X). Informed consent/assent was obtained, as appropriate.

### Participants

We included children and youth <18 years diagnosed with EoE treated with swallowed fluticasone or oral viscous budesonide for more than 1 month, over a 2 year period from 2011 to 2013. All included participants were diagnosed as per standard clinical and histological criteria [5]. They were refractory to proton pump inhibitor (PPI) therapy, and were symptomatic with dysphagia, abdominal pain, heartburn, or other symptoms consistent with EoE. All included participants had biopsy-proven EoE, defined as >15 eosinophils per high-powered field (hpf) from mucosal biopsies in any region of the esophagus obtained during esophagogastroduodenoscopy. Participants were followed for a minimum of 12 months or until August 2014 and were excluded if they had known adrenal insufficiency unrelated to GC therapy including ACTH deficiency due to hypothalamic or pituitary gland abnormalities, or a primary adrenal disorder. Participants who had received continuous oral or intravenous GC therapy for more than 2 weeks during the 6 month period preceding study enrollment were also excluded. The treatment protocol evaluated in our study represented a new standard of care for our EoE clinic and therefore all patients meeting the above criteria were enrolled consecutively.

### Treatment protocol

Participants were treated with oral viscous budesonide 1 mg (for children <5 feet tall) or 2 mg (for children/youth ≥5 feet tall) mixed with Splenda^®^ to form a viscous suspension (approximately 0.5 mg/2 mL) [11]. If they declined viscous budesonide, they were offered therapy with swallowed fluticasone. Fluticasone dose was 125 mcg/puff (for children <5 feet tall) or 250 mcg/puff (for children/youth ≥5 feet tall), swallowed once daily. Treatment at full dose was continued for 3 months, and then tapered by giving the same dose every other day for 1 month, followed by discontinuation of the swallowed topical GC therapy.

As part of our standard clinical practice guidelines, all patients diagnosed with AS are instructed to administer stress doses of GCs until resolution of AS (normalization of the LDST). Management of AS is outlined in Appendix Table 3.

### Assessment of adrenal axis functioning

A first morning cortisol level (drawn between 08:00 and 09:00) and LDST were arranged for 2 weeks (±3 days) following the final swallowed topical GC dose. First morning cortisol levels and LDST were performed in the Medical Day Unit at CHEO. If abnormal on initial testing, LDST was repeated 2 months following discontinuation of swallowed topical GC therapy, and every 3 months thereafter until resolution of AS (normalization of LDST). The duration of AS was defined as the time from the initial abnormal LDST to normalization of the LDST (>500 nmol/L).

### Laboratory investigations

Serum cortisol levels were measured by competitive binding immunoenzymatic assay (Beckman Coulter). First morning cortisol levels were measured in the serum on samples obtained between 08:00 and 09:00. A cortisol value of >185 nmol/L was considered to be normal; this normal value corresponds to our hospital biochemistry laboratory normal range for morning cortisol.

To perform the LDST, a peripheral intravenous line was established and 1 mcg of IV cosyntropin was administered (Cortrosyn, Amphastar Pharmaceuticals Inc.). Sampling of serum cortisol levels was done at 15, 30 and 60 min after cosyntropin administration. A normal LDST result was defined as a cortisol level peak >500 nmol/L [12, 13]. AS was defined as an abnormal LDST (cortisol peak <500 nmol/L) [12, 13].

### Statistical analysis

Descriptive statistics were calculated and reported as medians with interquartile range (IQR) or means with standard deviation (SD) where appropriate. Diagnostic accuracy of first morning cortisol was reported compared to the reference standard LDST (using 500 nmol/L as the cutoff value) by calculating sensitivity, specificity, positive predictive value (PPV), negative predictive value (NPV), and likelihood ratios (LR) with 95 % confidence intervals (CI). Agreement between first morning cortisol and LDST to identify those with AS was calculated using Cohen’s Kappa statistic (±standard error). The receiver operating characteristic (ROC) curve was used to determine the best cutoff of first morning cortisol to differentiate those with normal and abnormal LDST. Statistical analyses were performed using SPSS version 22 (IBM, Armonk, NY).

## Results

### Baseline characteristics

**Fig. 1:**
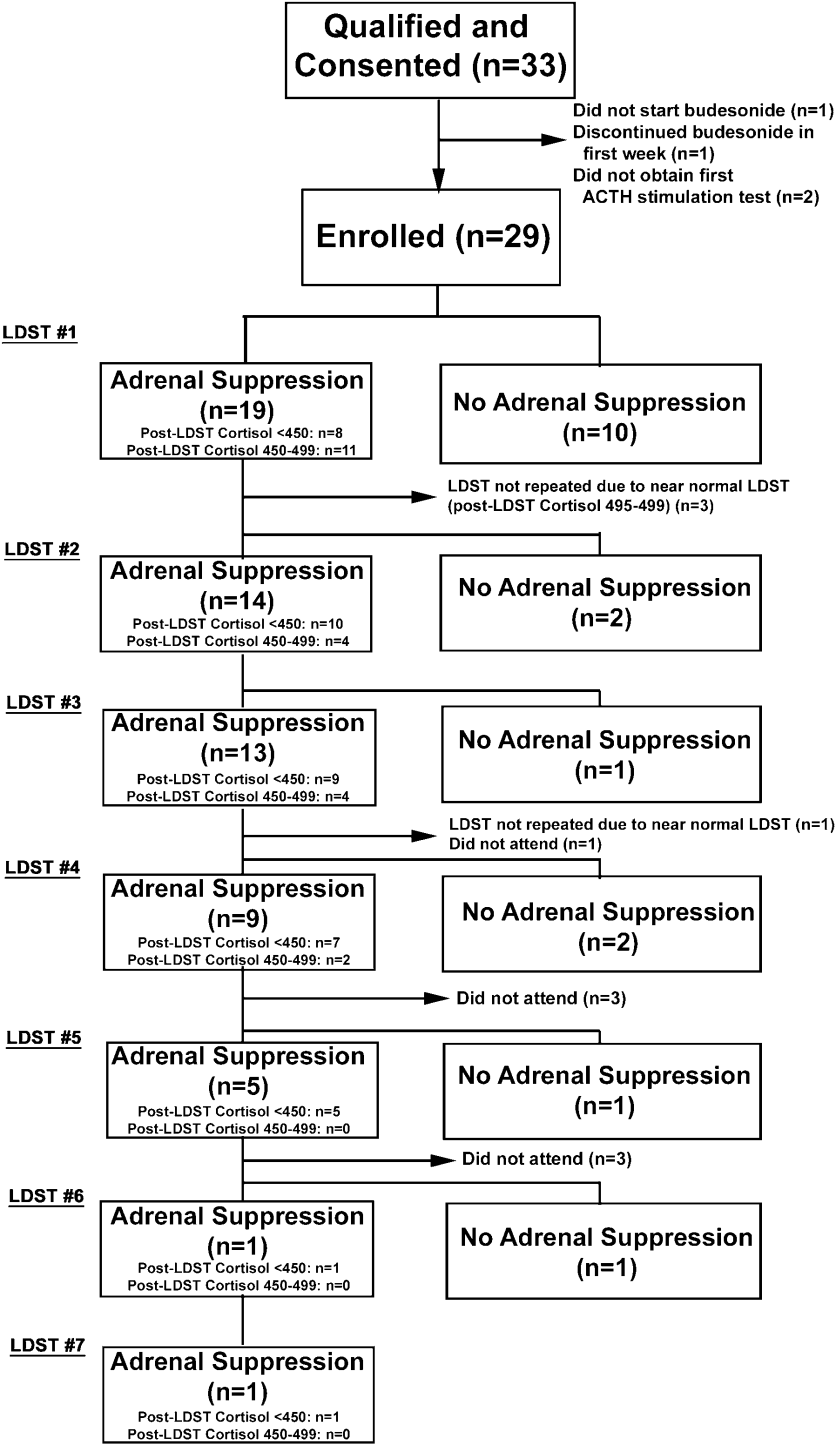
Study flow diagram

Characteristics of enrolled participants are outlined in Table 1. Thirty-three patients met the study inclusion criteria and consented to enrollment; 29 patients were enrolled (Fig. 1). Of the 29 study participants, 26 (89.7 %) were treated with oral viscous budesonide, and three (10.3 %) were treated with swallowed fluticasone. Thirteen participants (44.8 %) were receiving other forms of GCs (Table 1). None of the additional forms of GCs were systemic.

**Table 1:** Descriptive characteristics of study cohort

Characteristic	
Total number of patients analyzed	29
Female, N (%)	7 (24.1)
Median age (y) at diagnosis with EoE (IQR)	13.2 (6.4)
Median age (y) at study enrollment (IQR)	14.1 (4.6)
Features of GC therapy	
Duration of GC therapy (days ± SD)	104.6 ± 23.7
Oral viscous budesonide, N (%)	26 (89.7)
Swallowed fluticasone, N (%)	3 (10.3)
Concomitant conditions, N (%)	
Asthma	21 (72.4)
Allergies^a^	27 (93.1)
Eczema	10 (34.5)
Additional forms of GC therapy, by patient, N (%)	
Total number of patients receiving additional forms	13 (44.8 %)
Inhaled corticosteroids^b^	11 (37.9 %)
Intranasal	1 (3.4 %)
Topical	2 (6.9 %)

*EoE* eosinophilic esophagitis, *ICS* inhaled corticosteroids, *IQR* interquartile range, *GC* glucocorticoid, *N* number, *SD* standard deviation, *y* years

^a^A diagnosis of allergy was made based on skin prick testing for food and/or environmental allergies by an allergist

^b^Most participants had a history of infrequent ICS use during inter-current infection only. Two participants were being actively treated with daily moderate ICS doses (Flovent 250 mcg daily and Alvesco 400 mcg daily)

### Initial testing for adrenal suppression

**Fig. 2:**
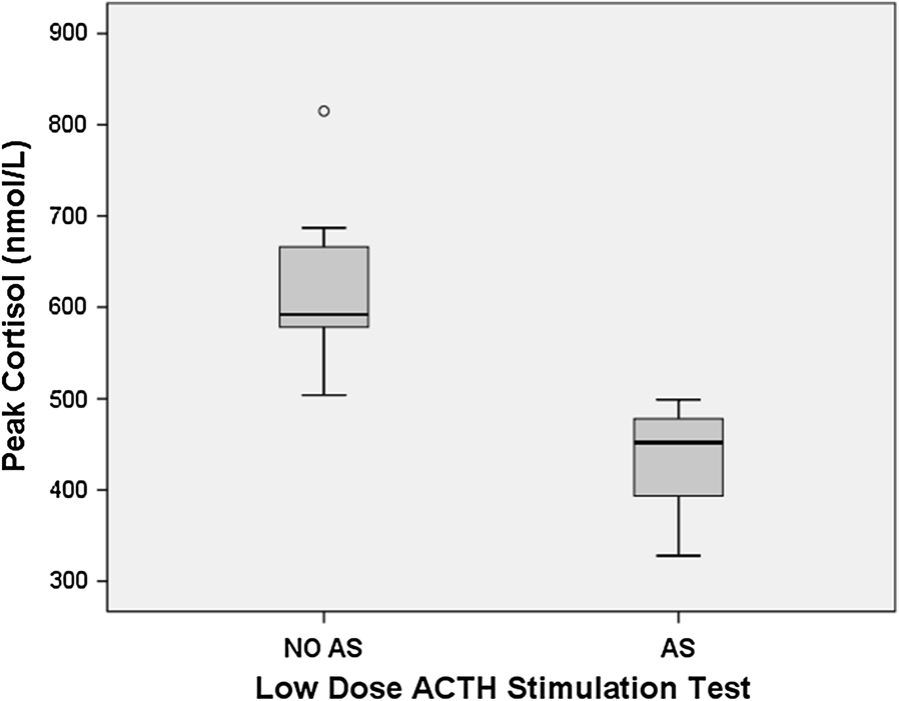
Peak cortisol level on ACTH stimulation test in adrenal suppression (AS) vs no adrenal suppression (NO AS) group

Two weeks following discontinuation of GC therapy, 19 (65.5 %) participants were found to have AS, as defined by a LDST peak <500 nmol/L on initial testing (Fig. 2). Of those, 11 of 19 (57.9 %) had a borderline LDST result (peak cortisol 450–499 nmol/L), and 8 of 19 (42.1 %) had a LDST peak <450 nmol/L (Fig. 1). All but one of the participants with AS were treated with budesonide. On exploratory univariate analysis, there were no significant predictors of AS, including concomitant ICS use (Table 2).

**Table 2:** Potential predictors of adrenal suppression

	No AS	AS	p value
Female sex (N, %)	3/10 (30 %)	4/19 (21.1 %)	0.66^a^
Age (median, IQR)	15.15 (4.75)	13.81 (4.66)	0.31^b^
Duration of GC therapy (days)	113.5 ± 30.44	99.8 ± 18.44	0.22^c^
BMI	18.2 ± 2.54	19.3 ± 3.15	0.33^c^
Concurrent use of ICS therapy (intermittent or continuous)	2/10 (20 %)	9/19 (47.3 %)	0.23^a^
Concurrent use of ICS therapy (continuous only)	1/10 (10 %)	5/19 (26.3 %)	0.63^a^

*AS* adrenal suppression, *BMI* body mass index, *GC* glucocorticoid, *ICS* inhaled corticosteroids, *IQR* interquartile range, *N* number

^a^Fisher’s Exact test

^b^Wilcoxon-Mann–Whitney test

^c^T test (equal variances not assumed)

**Fig. 3:**
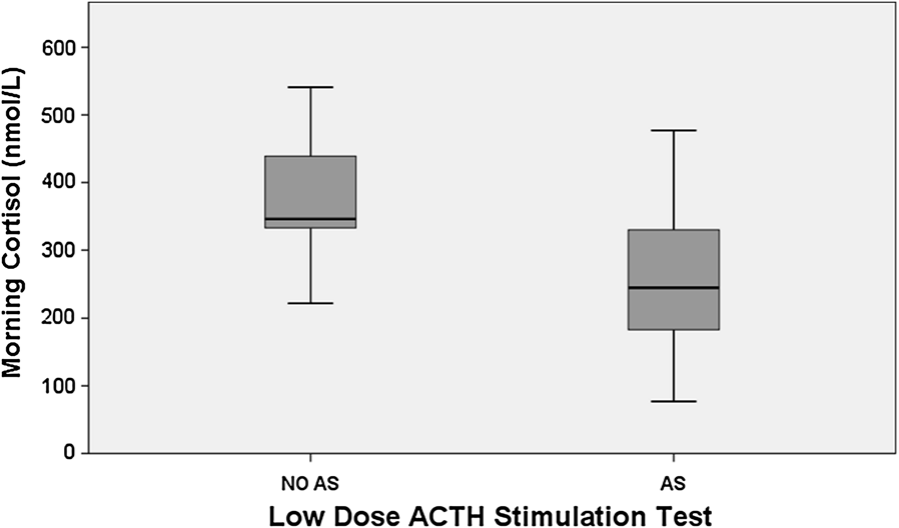
First morning cortisol value in adrenal suppression (AS) vs no adrenal suppression (NO AS) group

Five of the 19 participants (26.3 %) with confirmed AS had an abnormal first morning cortisol (<185 nmol/L) (Fig. 3). There was only slight agreement between first morning cortisol and LDST to define AS (Kappa 0.198 ± 0.09, P = 0.092). The diagnostic accuracy of abnormal first morning cortisol (<185 nmol/L) to identify AS (defined by an abnormal LDST) was as follows: sensitivity 26.3 % (95 % CI 45.7–81.4 %), specificity 100 % (95 % CI 65.5–100 %), PPV 100 % (95 % CI 46.3–100 %), NPV 37.5 % (95 % CI 19.6–59.2 %), LR+ infinite, LR− 0.75 (95 % CI 0.58–0.97). As defined by the ROC curve, the best cutoff of first morning cortisol to define AS was 323 nmol/L, which differentiated AS participants from those without AS with sensitivity 70.0 % (95 % CI 45.7–87.2 %), specificity 77.8 % (95 % CI 40.2–96.1 %), PPV 87.5 % (95 % CI 60.4–97.8 %), NPV 53.8 % (95 % CI 26.1–79.6 %), LR+ 3.15 (95 % CI 0.90–11.05), LR− 0.39 (95 % CI 0.19–0.80).

### Longitudinal testing for sustained adrenal suppression

Median duration of AS was 42.7 (IQR 11.1–60.6) weeks. AS lasted >365 days in at least 5 of 19 (26.3 %) participants; all of which had a LDST peak <450 nmol/L. Details of longitudinal testing are depicted in Fig. 1. There were no reported cases of adrenal crisis during the study period.

### Availability of data and materials

An additional Excel file shows the dataset on which the conclusions of the manuscript rely (Additional file 1).

## Discussion

The findings of our study demonstrate that children are at significant risk of prolonged AS following treatment with swallowed topical glucocorticoids for the treatment of EoE. Almost two-thirds of the children in our study had AS 2 weeks following treatment with a median duration of 42 weeks; several had persistent AS beyond 1 year. Our prospective study provides an important contribution to our understanding of AS in the EoE population, demonstrating that this potential side effect is not only a concern while on treatment, but continues following discontinuation of swallowed glucocorticoid therapy. In addition, we demonstrated that first morning cortisol was not a sensitive test for AS when compared with the LDST.

Previous studies examining AS in children treated with swallowed topical GC have resulted in mixed conclusions. A national surveillance study of symptomatic AS from any form of GC reported that 2 of 46 children were treated with swallowed topical GC, suggesting that the EoE population might be at increased risk [14]. A recent retrospective study also suggested that children who were *being actively treated* with swallowed budesonide for EoE were at increased risk of AS [15]. Our study confirmed these warnings. In contrast to the previous study [15], we tested 2 weeks after discontinuation of topical GC therapy to rule out acute transient AS that would be expected while receiving GC therapy.

Our findings differed from a recent prospective study that examined 14 children receiving swallowed topical GCs concluding that there was no significant effect on the adrenal axis [16]; the conflicting results were likely secondary to the testing modality chosen to define AS. Morning cortisol was used to evaluate the HPA axis [15]; we demonstrated that this test has very poor sensitivity for AS indicating that dynamic testing should be considered instead.

Our findings suggest that an abnormal first morning cortisol (<185 nmol/L) is highly specific for adrenal suppression in the EoE population treated with swallowed topical GC therapy but that sensitivity is poor. The cutoff of 185 nmol/L is significantly higher than cutoffs previously reported in the literature [17–19] and likely reflects a higher pre-test probability of AS in the EoE population. While it is important for clinicians to be aware that cortisol thresholds vary slightly between platforms and cannot be directly compared, it is unlikely that this accounts for our results [18, 20]. Our findings are consistent with previous studies in other populations, suggesting that the LDST with sensitivity and specificity around 90 % [12, 21], is required in the evaluation of AS unless first morning cortisol values are very high or very low [19, 22].

Potential systemic side effects of ICS, including AS, occur when the swallowed portion escapes inactivation by first-pass metabolism in the liver and enters the systemic circulation [4]. A recent study in adults with EoE concluded that active disease may result in reduced elimination of budesonide via CYP3A, compared to healthy adults, suggesting a possible increased risk of AS in the EoE population [23].

Adrenal crisis during surgery and illness in patients with adrenal insufficiency who have not received stress doses of GC therapy is well documented [2, 8]. General anesthesia is also a potential stressor and is a consideration in patients with EoE undergoing gastrointestinal endoscopy. A recent CSACI position statement provides an approach to screening and management of potential AS in children treated with ICS for asthma [6]. Our study suggests that clinicians should be aware of the potential for AS in all children being treated with swallowed topical corticosteroids and consider GC stress dosing for significant physiological stress until significant abnormalities on LDST are ruled out. If there are unexplained non-specific symptoms (i.e. fatigue, malaise, nausea, vomiting, abdominal pain, headache) or poor growth, then testing of the HPA axis and consultation with a pediatric endocrinologist should be sought. In addition, a pediatric endocrinologist should be consulted for individuals with prolonged AS based on LDST.

A possible limitation of our study is the absence of LDST prior to initiation of swallowed topical GC therapy. Almost half of the study population received other forms of GC therapy for treatment of asthma, eczema and allergic rhinitis; yet none were receiving regular high doses of ICS. Fifty three percent of children with AS in our study did not receive concomitant GC therapy, demonstrating that swallowed topical GCs can cause AS independently. Although concomitant GC use was not demonstrated to be a risk factor for AS, our study may not have been powered to detect this. The use of the lowest effective doses of all forms of GC may help in the prevention of AS [3].

This study was limited by the small number of enrolled participants; the univariate analysis of risk factors for AS was exploratory only as our study may not have been powered to detect predictors. While 27.6 % of our children had significantly abnormal LDST results, 57 % of children in the AS group had a “borderline” cortisol peak on LDST (450–499 nmol/L), the clinical significance of which is difficult to interpret. To our knowledge, there is no literature that has examined the risk of symptomatic AS in individuals with mildly abnormal LDST. As such, all participants with documented AS were treated with stress doses of GC therapy for illness, surgery or injury as per standard of care [1].

In conclusion, almost two-thirds of the children/youth in our EoE cohort had evidence of AS detected by LDST following discontinuation of swallowed topical GC therapy. Twenty seven percent of the subjects demonstrated significant abnormalities on testing, indicating that AS is a true concern in EoE. When present, AS was persistent and prolonged. Unrecognized AS can lead to adrenal crisis with significant morbidity and even mortality [1, 2]. We recommend consideration of GC stress dosing during significant physiological stress (moderate to severe illness or injury, general anesthesia or surgery) in children/youth being treated with swallowed topical GC for EoE both during and after discontinuation of their swallowed topical GC therapy and until significant abnormalities on LDST are ruled out. Borderline results on LDST need to be evaluated on an individual basis; future study addressing the risk in these patients is needed. This study highlights the essential need for further education of EoE patients, families and the health care team about the symptoms of AS and the management of illness in the context of AS.

## Appendix

See Table 3.

**Table 3:** Management of AS—recommendations given to patients/families of children with EoE at CHEO

Stress dosing recommended to all patients until normalization of LDST	
Mild illness or fever	20 mg/m^2^/day hydrocortisone equivalent, divided BID or TID
Fever >38.5 or vomiting	30 mg/m^2^/day hydrocortisone equivalent, divided TID
Severe illness or injury OR unable to tolerate oral GC during illness	ER for IV GC therapy
Surgery or procedure with anesthesia	Endocrinology consulted for IV GC dosing
Patients with first morning cortisol <185 nmo/L	
Daily glucocorticoid therapy	10 mg/m^2^/day q am
Illness/surgery/injury	Stress dosing as outlined above

*BID* twice daily, *TID* three times daily, *ER* emergency department, *IV* intravenous, *q am* daily morning

## Additional file

10.1186/s13223-016-0154-9 Eosinophilic esophagitis adrenal suppression dataset.
